# Supplementary material for: A study on assessing the toxic effects of ethyl paraben on rohu (Labeo rohita) using different biomarkers; hemato-biochemical assays, histology, oxidant and antioxidant activity and genotoxicity
Source: PLoS One. 2024 May 6;19(5):e0302691. doi: 10.1371/journal.pone.0302691 (PMC11073686; doi:10.1371/journal.pone.0302691)
Supplement: Attachment — Submitted filename: Response to Reviewers.docx [file pone.0302691.s005.docx]

**A study on assessing the toxic effects of ethyl paraben on rohu (*Labeo rohita*) using different biomarkers; Hemato-biochemical assays, Histology, Oxidant and Antioxidant activity and Genotoxicity**

Dear Editor-in-Chief

I hope this letter finds you well. I am writing to inform you about the corrections and revisions we have made to our manuscript (PONE-D-23-29563), titled " **A study on assessing the toxic effects of ethyl paraben on rohu (*Labeo rohita*) using different biomarkers; Hemato-biochemical assays, Histology, Oxidant and Antioxidant activity and Genotoxicity**" which was submitted to PLOS ONE. We would like to express our sincere gratitude for the insightful comments and suggestions provided by the reviewers. We have carefully considered all the feedback and made significant improvements to our manuscript based on their valuable input.

**Respond to comments of reviewers:**

| **Comments** | | **Responses** |
| --- | --- | --- |
| 01 | Please ensure that your manuscript meets PLOS ONE's style requirements, including those for file naming. | Revised and corrected |
| 02 | Please clarify the sources of funding (financial or material support) for your study. List the grants or organizations that supported your study, including funding received from your institution, .... | The authors received no specific funding for this work.” |
| 03 | Please remove any funding-related text from the manuscript. | Revised, edited and corrected |
| 04 | In your Data Availability statement, you have not specified where the minimal data set underlying the results described in your manuscript can be found. PLOS defines a study's minimal data set as the underlying data used to reach the conclusions drawn in the manuscript and any additional data required to replicate the reported study findings in their entirety. | All data set are present in submitted manuscript. Raw data from this study are available upon request. |
| 05 | We note that you have indicated that data from this study are available upon request. PLOS only allows data to be available upon request if there are legal or ethical restrictions on sharing data publicly. | No legal or ethical restrictions on sharing data publicaly. |
| 06 | Please ensure that you refer to Figure 3 in your text as, if accepted, production will need this reference to link the reader to the figure. | corrected |
| 07 | Please include a caption for figure 7. | corrected |
| 08 | Please upload a copy of Figure 7, to which you refer in your text on page 19 in PDF submission. If the figure is no longer to be included as part of the submission please remove all reference to it within the text. | Revised, edited and corrected |
| 09 | We note you have included a table to which you do not refer in the text of your manuscript. Please ensure that you refer to Table 2 in your text; if accepted, production will need this reference to link the reader to the Table. | Revised, edited and corrected |

| **Respond to comments of Reviewer #1** | | **Responses** |
| --- | --- | --- |
| 01 | The manuscript needs language editing.  The introduction section should be improved.  The methodology needs more details.  The aim of work not clear | Revised and corrected |
| 02 | Abstract section  -line 18, insert a full stop after (Products)  -line 19 to 21 rephrase  -the abstract should be informative, the scientific name of fish should be added, number of fish used and their average weight, number of fish groups, number of replicates, and the experimental duration. | Revised and corrected |
| 03 | The methodology should mention briefly in the abstract.  -Add a space between T0(0.00 µg/L). | Revised and corrected |
| 04 | the full name of all abbreviations written in the abstract should be written  - (Due to higher oxidative enzymes activity antioxidant enzymes potential to overcome their stress activity reduced) this sentence should be deleted, no explanations were written in the abstract | Revised and corrected |
| 05 | which level of ethyl paraben that produced the results written in the abstract |  |
| 06 | Keywords: remove the word, aquatic toxicology | Revised and corrected |
| 07 | Introduction section  -the introduction section lack the information about the toxic impact of paraben on fish tissue, health  Also the residues of such paraben in water bodies, how paraben gain access to the water bodies. The uses of paraben in agriculture practices  -the significance of the studied fish (rohu, Labeo rohita) should be added in the introduction section  Why the authors used this fish as toxicological model  -the aim of work not clear because the problem not clarified well in the introduction section | Revised and corrected |
| 08 | Material and method section  1-line 91, replace made with assigned  2-number of fish and weight should be mentioned  3-what is the LC50 value for ethyl parapen, why authors evaluated it for 48 hours not for 96 hours  4- What is the relation between the lC50 and the different doses of parapen used in the experiment?  5-line 109, add the dose of clove oil and reference  6-the number of samples should be written for each group in all methodology (blood samples, histological samples, ….etc)  7-the procedures of histological examinations is lack many details, so superficial  The details should be added  8- All abbreviations should be defined for the first time  9- For the statistical analysis, the normality test should be performed before the ANOVA | Revised and corrected |
| 09 | Results  1- All tables titles should be rephrased and rewritten  2- Table footnotes should be added and the full names of all measured parameters should be inserted in the footnote  3- Line 196, the author written (propyl paraben), the authors used ethyl paraben or propyl paraben  4- All data in the figures and tables should be carefully revised by the authors  Discussion section  This part should be revised and improved  Line 320-321 why written in italic form  Many language errors are present | Revised and corrected |
| 10 | Conclusion section: this section not present | Revised and corrected |

| **Respond to comments of Reviewer #2** | | **Responses** |
| --- | --- | --- |
| 01 | The abstract has not been written in a clear and concise manner. For example the paragraph starting with ethyl paraben induced oxidative .....; is not stating the findings clearly. | Revised and corrected |
| 02 | There is reference to genotoxicity in the abstract. However, it is not clear which marker for genotoxicity was used to arrive at this conclusion. If this was based on the COMET Assay, then indicate that "based on the comet assay, | Revised and corrected |
| 03 | Under methods: it is not clear how the dosages of 2000, 4000, and 6000 were chosen. Is this based on actual estimated concentrations of paraben in contaminated waterways? How much paraben is fish exposed to in water? Do they bio-accumulate in fish tissues? What steps were taken to ensure that prior exposure to paraben and related chemicals did not occur in regard to fish used for the study? This needs to come out clearly. | As per previous work, LC50 was determined. So, according to this, LC50 doses were divided into ½, 1/3, and ¼. These doses were actually based on laboratory work, and their estimation in natural or contaminated water is under process. We did not work on the estimation of paraben in fish. Fish were taken from fresh water fish form, where treated water was used. |
| 04 | Quite significantly, the manuscript has recurring grammar issues, and poor prose/sentence construction. For example, the sentence that reads..." all experiments were formed.... The word "formed" is quite out of place. This is repeated throughout (line 75). Experiments are "conducted or performed" etc... Do not use don't..., rather use do not etc... | Revised and corrected |
| 05 | Did the study investigate levels of glutathione or glutathione reductase? In the results, the sub-title is GSH, which is used to abbreviate glutathione and not glutathione reductase. In the discussion and figures, references have been made in regard to antioxidants including glutathione (GSH). Where does glutathione reductase come in line 140-144? |  |
| 06 | Hematology data has been presented in a table format. This is not correct. Dose response studies are best presented using line graphs to easily decipher the trend. This observation applies to antioxidant analysis (GSH, TBARS, ROS, SOD, CAT etc..) | Revised and corrected |
| 07 | The data on antioxidant assay is quite confusing for liver/kidney. ROS is going up, consistent with TBARS; but antioxidant enzymes that are meant to be up-regulated in response to exposure to chemical toxicants such as paraben are actually being down-regulated. This is a paradox. Note that there is a dose-dependent rise in ROS. Antioxidants are expected to rise in concert to the rise in ROS. For this study, antioxidants are going down as the dose of paraben goes up. However, many studies have demonstrated depletion of GSH levels in response to overwhelming chemical-induced oxidative stress. This may explain the decrease in GSH. Not sure about the SOD, CAT etc..,. This scenario may be possible when there is overwhelming oxidative stress, depleting all the antioxidants. Clarify this phenomenon. Notably, liver function markers are all up-regulated, signaling severe paraben-driven liver injury. | Explained in line 345-379 |
| 08 | Data for hematology shows evidence for paraben-driven significant up-regulation of platelets. This elevation will have serious implications for blood-related disorders like DVT and patients who may be on blood thinning medications. This aspect needs to be discussed more broadly in the discussion section to highlight implications in various circumstances. Infact, RBCs and HGB will be expected to be down-regulated due to a possible paraben-driven impediment to hematopoiesis. | Explained in line 285-290 |
| 09 | It is not clear why paraben will result in elevation of HGB, MCV, MCHC and platelets. | Revised and corrected |
| 10 | Notably, at low dosages, the effect of paraben on blood indices was low, validating the premise of this study. This, probably needs to come out well in the discussion. | Revised and corrected |
| 11 | It is very important that the grammar issues are corrected, and the manuscript is written carefully in a concise and logical manner using appropriate english | Revised and corrected |
| 12 | There is need for a more concise title tied to the major findings of the study. | Revised and corrected |

Thank you for your time and consideration. We eagerly await your decision regarding the acceptance of our manuscript.

Regards

Corresponding author
