# Supplementary material for: A study on assessing the toxic effects of ethyl paraben on rohu (Labeo rohita) using different biomarkers; hemato-biochemical assays, histology, oxidant and antioxidant activity and genotoxicity
Source: PLoS One. 2024 May 6;19(5):e0302691. doi: 10.1371/journal.pone.0302691 (PMC11073686; doi:10.1371/journal.pone.0302691)
Supplement: Attachment — Submitted filename: Response to Reviewers.docx [file pone.0302691.s006.docx]

**A study on assessing the toxic effects of ethyl paraben on rohu (*Labeo rohita*) using different biomarkers; Hemato-biochemical assays, Histology, Oxidant and Antioxidant activity and Genotoxicity**

Dear Editor-in-Chief

I hope this letter finds you well. I am writing to inform you about the corrections and revisions we have made to our manuscript (PONE-D-23-29563), titled " **A study on assessing the toxic effects of ethyl paraben on rohu (*Labeo rohita*) using different biomarkers; Hemato-biochemical assays, Histology, Oxidant and Antioxidant activity and Genotoxicity**" which was submitted to PLOS ONE. We would like to express our sincere gratitude for the insightful comments and suggestions provided by the reviewers. We have carefully considered all the feedback and made significant improvements to our manuscript based on their valuable input.

**Respond to comments of reviewers:**

| **Respond to comment of Reviewer #2** | | **Response** |
| --- | --- | --- |
| 01 | I gave detailed review proposals in my first analysis of this work. The authors have made reasonable effort to correct the figure for dose response studies as i guided. However, the grammar issues have not been addressed across the board starting with the abstract, through to the discussion section. A lot of work needs to be done here. | Revised and corrected |

Thank you for your time and consideration. We eagerly await your decision regarding the acceptance of our manuscript.

Regards

Corresponding author

Dr.Khurram Shahzad

Assistant Professor of Zoology

University of Okara, Pakistan

dr.khurram@uo.edu.pk

ORCID: https://orcid.org/0000-0001-9152-3736
