# Supplementary material for: A study on assessing the toxic effects of ethyl paraben on rohu (Labeo rohita) using different biomarkers; hemato-biochemical assays, histology, oxidant and antioxidant activity and genotoxicity
Source: PLoS One. 2024 May 6;19(5):e0302691. doi: 10.1371/journal.pone.0302691 (PMC11073686; doi:10.1371/journal.pone.0302691)
Supplement: Attachment — Submitted filename: Responce to reviewer.docx [file pone.0302691.s007.docx]

| **Comment** | **Answer** |
| --- | --- |
| If applicable, we recommend that you deposit your laboratory protocols in [protocols.io](http://protocols.io/) to enhance the reproducibility of your results. | Not applicable |
| Please review your reference list to ensure that it is complete and correct. If you have cited papers that have been retracted, please include the rationale for doing so in the manuscript text, or remove these references and replace them with relevant current references. Any changes to the reference list should be mentioned in the rebuttal letter that accompanies your revised manuscript. | We have reviewed all references. No cited paper has been retracted. |
| While revising your submission, please upload your figure files to the Preflight Analysis and Conversion Engine (PACE) digital diagnostic tool, | Corrected and submitted in supporting information |
