# Supplementary material for: A study on assessing the toxic effects of ethyl paraben on rohu (Labeo rohita) using different biomarkers; hemato-biochemical assays, histology, oxidant and antioxidant activity and genotoxicity
Source: PLoS One. 2024 May 6;19(5):e0302691. doi: 10.1371/journal.pone.0302691 (PMC11073686; doi:10.1371/journal.pone.0302691)
Supplement: Attachment — Submitted filename: Response to Reviewers.docx [file pone.0302691.s008.docx]

**A study on assessing the toxic effects of ethyl paraben on rohu (*Labeo rohita*) using different biomarkers; Hemato-biochemical assays, Histology, Oxidant and Antioxidant activity and Genotoxicity**

Dear Editor-in-Chief

I hope this letter finds you well. I am writing to inform you about the corrections and revisions we have made to our manuscript (PONE-D-23-29563R3), titled " **A study on assessing the toxic effects of ethyl paraben on rohu (*Labeo rohita*) using different biomarkers; Hemato-biochemical assays, Histology, Oxidant and Antioxidant activity and Genotoxicity**" which was submitted to PLOS ONE. We would like to express our sincere gratitude for the insightful comments and suggestions provided by the reviewers. We have carefully considered all the feedback and made significant improvements to our manuscript based on their valuable input.

**Respond to comments of reviewers:**

| **Respond to comment** | | **Response** |
| --- | --- | --- |
| 01 | I have observed that your response to the reviewers' comments was not addressed carefully, and the quality of English in your manuscript still needs improvement. | Revised and improved English. |
| 02 | Please review your reference list to ensure that it is complete and correct. If you have cited papers that have been retracted, please include the rationale for doing so in the manuscript text, or remove these references and replace them with relevant current references | We cross checked all references and we haven’t found any retracted reference. Please let us know any retracted reference. |
| 03 | While revising your submission, please upload your figure files to the Preflight Analysis and Conversion Engine (PACE) digital diagnostic tool. | Figures have been uploaded and resized according to plos requirements and uploaded as figure and raw data in supporting information title “figure. Rar”. |

Thank you for your time and consideration. We eagerly await your decision regarding the acceptance of our manuscript.

Regards

Corresponding author

Dr.Khurram Shahzad

Assistant Professor of Zoology

University of Okara, Pakistan

dr.khurram@uo.edu.pk

ORCID: https://orcid.org/0000-0001-9152-3736
