# Supplementary material for: A study on assessing the toxic effects of ethyl paraben on rohu (Labeo rohita) using different biomarkers; hemato-biochemical assays, histology, oxidant and antioxidant activity and genotoxicity
Source: PLoS One. 2024 May 6;19(5):e0302691. doi: 10.1371/journal.pone.0302691 (PMC11073686; doi:10.1371/journal.pone.0302691)
Supplement: Attachment — Submitted filename: reviewer comments to PONE-D-23-29563.docx [file pone.0302691.s004.docx]

The manuscript entitled (A study on assessing the toxic effects of ethyl paraben on rohu (*Labeo rohita*) using different biomarkers; Hemato-biochemical assays, Histology, Oxidant and Antioxidant activity and Genotoxicity) needs major changes before being published

**General comments:**

The manuscript needs language editing

The introduction section should be improved

The methodology needs more details

The aim of work not clear

**Specific comments**

**Abstract section**

-line 18, insert a full stop after (Products)

-line 19 to 21 rephrase

-the abstract should be informative, the scientific name of fish should be added, number of fish used and their average weight, number of fish groups, number of replicates, and the experimental duration.

The methodology should mention briefly in the abstract.

-Add a space between T0(0.00 µg/L).

-the full name of all abbreviations written in the abstract should be written

- (Due to higher oxidative enzymes activity antioxidant enzymes potential to overcome their stress activity reduced) this sentence should be deleted, no explanations were written in the abstract

-which level of ethyl paraben that produced the results written in the abstract

**Keywords:** remove the word, aquatic toxicology

**Introduction section**

-the introduction section lack the information about the toxic impact of paraben on fish tissue, health

Also the residues of such paraben in water bodies, how paraben gain access to the water bodies. The uses of paraben in agriculture practices

-the significance of the studied fish (rohu, Labeo rohita) should be added in the introduction section

Why the authors used this fish as toxicological model

-the aim of work not clear because the problem not clarified well in the introduction section

-add the following references in the introduction section, they will be useful

1- Ahmed, S.A., Ibrahim, R.E., Elshopakey, G.E., Khamis, T., Abdel-Ghany, H.M., Abdelwarith, A.A., Younis, E.M., Davies, S.J., Elabd, H., Elhady, M.J.F., Immunology, S., 2022. Immune-antioxidant trait, growth, splenic cytokines expression, apoptosis, and histopathological alterations of Oreochromis niloticus exposed to sub-lethal copper toxicity and fed thyme and/or basil essential oils enriched diets. 131, 1006-1018.

2- Ibrahim, R.E., El-Houseiny, W., Behairy, A., Mansour, M.F., Abd-Elhakim, Y.M., 2019. Ameliorative effects of Moringa oleifera seeds and leaves on chlorpyrifos-induced growth retardation, immune suppression, oxidative stress, and DNA damage in Oreochromis niloticus. Aquaculture 505, 225-234.

3- Mohamed, W.A., El-Houseiny, W., Ibrahim, R.E., Abd-Elhakim, Y.M., 2019. Palliative effects of zinc sulfate against the immunosuppressive, hepato-and nephrotoxic impacts of nonylphenol in Nile tilapia (Oreochromis niloticus). Aquaculture 504, 227-238.

4- Ibrahim, R.E., Elbealy, M.A., Salem, G.A., Abdelwarith, A.A., Younis, E.M., Wagih, E., Elkady, A.A., Davies, S.J. and Rahman, A.N.A., 2023. Acute mancozeb-fungicide exposure induces neuro-ethology disruption, health disorders, and immune-oxidative dysfunction in Nile tilapia (Oreochromis niloticus). *Aquatic Toxicology*, *261*, p.106630.

5- Ibrahim, Rowida E., Heba I. Ghamry, Saed Ayidh Althobaiti, Daklallah A. Almalki, Medhat S. Shakweer, Mona A. Hassan, Tarek Khamis, Heba M. Abdel-Ghany, and Shaimaa AA Ahmed. "Moringa oleifera and Azadirachta indica Leaves enriched diets mitigate chronic oxyfluorfen toxicity induced immunosuppression through disruption of pro/anti-inflammatory gene pathways, alteration of antioxidant gene expression, and histopathological Alteration in Oreochromis niloticus." *Fishes* 8, no. 1 (2022): 15.

**Material and method section**

1-line 91, replace made with assigned

2-number of fish and weight should be mentioned

3-what is the LC50 value for ethyl parapen, why authors evaluated it for 48 hours not for 96 hours

4- What is the relation between the lC50 and the different doses of parapen used in the experiment?

5-line 109, add the dose of clove oil and reference

6-the number of samples should be written for each group in all methodology (blood samples, histological samples, ….etc)

7-the procedures of histological examinations is lack many details, so superficial

The details should be added

8- All abbreviations should be defined for the first time

9- For the statistical analysis, the normality test should be performed before the ANOVA

**Results**

1- All tables titles should be rephrased and rewritten
2- Table footnotes should be added and the full names of all measured parameters should be inserted in the footnote
3- Line 196, the author written (propyl paraben), the authors used ethyl paraben or propyl paraben
4- All data in the figures and tables should be carefully revised by the authors

**Discussion section**

This part should be revised and improved

Line 320-321 why written in italic form

Many language errors are present

**Conclusion section:** this section not present
